# Acoustic Moiré Flat Bands in Twisted Heterobilayer Metasurface

**DOI:** 10.1002/adma.202418839

**Published:** 2025-05-09

**Authors:** Shida Fan, Chenglin Han, Kuan He, Liang Bai, Li‐Qun Chen, Huaitao Shi, Chen Shen, Tianzhi Yang

**Affiliations:** ^1^ School of Mechanical Engineering and Automation Northeastern University Shenyang 110819 China; ^2^ School of Science Harbin Institute of Technology Shenzhen 518055 China; ^3^ School of Mechanical Engineering Shenyang Jianzhu University Shenyang 110168 China; ^4^ Department of Mechanical Engineering Rowan University Glassboro NJ 08028 USA

**Keywords:** acoustic magic angle, heterobilayer acoustic metasurfaces, robustness, topological transitions

## Abstract

Twisted bilayer systems enable a fundamental platform for engineering novel physical phenomena, such as topological phase transitions, polaritons, flat bands, and superconductivity. However, previous reports mainly focused on homobilayer structures, where each layer has identical configurations. In this work, a twisted heterobilayer (tHB) metasurface with strong anisotropy is presented. It is shown that hybridizing the dispersion curves of two different profiles significantly enhances the application potential of bilayer structures. It is observed that when two stacked heterogeneous metasurfaces are rotated to a specific angle, a topological phase transition occurs, which is accompanied by hyperbolic to elliptical wave propagation characteristics. At a specific “magic angle”, the dispersion curves of the two layers merge into a flat band with drastically reduced diffraction, enabling sound waves to propagate with minimal energy dissipation. Furthermore, the performance of the tHB system is robust to defects or disorders, manifested by minimal changes before and after introducing line defects into the layers. Our findings overcome the limitations of homogeneous metasurface and provide new ideas for controlling sound‐material interactions.

## Graphical abstract

This research proposes a heterobilayer moiré acoustic metasurface using two distinct lattice structures. The heterobilayer design contributes to divergent dispersion curves for the upper and lower layers and allows for greater flexibility in tuning their hybridization through interlayer twisting. This metasurface enables tunable magic angles and topological states, significantly broadening the potential applications of acoustic wave manipulation.

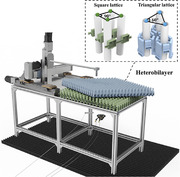

## 1 Introduction

Recently, significant advancements have been made in wave manipulation through the study of twisted bilayer metasurfaces.^[^ ^1^, ^2^, ^3^ ^]^ This has led to the discovery of novel phenomena such as hyperbolic metasurfaces,^[^ ^4^, ^5^, ^6^, ^7^ ^]^ flat‐band superconductors,^[^ ^8^, ^9^ ^]^ invisibility cloaks,^[^ ^10^, ^11^, ^12^ ^]^ and interlayer magnetism.^[^ ^13^ ^]^ The realization of these effects is primarily based on the twisted stacking of 2D materials,^[^ ^14^, ^15^, ^16^, ^17^ ^]^ where the twist angle controls the moiré superlattice and interlayer hybridization.^[^ ^18^, ^19^ ^]^ Originating from electromagnetics, many of these concepts have been successfully applied to acoustics thanks to the analogy of wave propagation properties, thus enabling the manipulation of sound waves.^[^ ^20^, ^21^, ^22^, ^23^ ^]^

However, the scalar nature of sound pressure^[^ ^24^, ^25^ ^]^ limits opportunities for realizing certain wave propagation phenomena in acoustics. For example, the concept of hyperbolic metasurfaces was first introduced in photonic crystals,^[^ ^26^, ^27^, ^28^, ^29^, ^30^, ^31^ ^]^ with the goal of achieving extreme anisotropy in materials. The required extreme anisotropy is fulfilled by manipulating the dielectric constant and permeability to exhibit anisotropic properties based on the vector nature of electromagnetic waves.^[^ ^32^, ^33^, ^34^ ^]^ Such an analogy does not exist for sound waves, making it challenging for their realization. To date, several methods have been developed to overcome these limitations, including solid‐fluid coupling,^[^ ^35^ ^]^ interlayer coupling breaking symmetry,^[^ ^36^ ^]^ and 3D chiral mechanical metamaterials,^[^ ^37^ ^]^ though these approaches still face certain challenges. Li and Alu^[^ ^38^ ^]^ proposed a non‐local hyperbolic metasurface based on non‐local unit cells,^[^ ^39^, ^40^, ^41^, ^42^ ^]^ which takes into account multidirectional coupling effects and energy tunneling, laying the groundwork for hyperbolic sound wave propagation. Their result suggests that in the hyperbolic regime, by changing the twist angle, the metasurface can undergo a Lifshitz topological transition,^[^ ^43^, ^44^ ^]^ where the dispersion curves transition from an open hyperbolic topology to a closed elliptical topology without changing the frequency. This topological transition's invariant corresponds to the non‐accidental crossing points (*N_ACPS_*) in the dispersion curves of moiré bilayer metasurfaces. The topological transition was proposed by Yves et al^[^ ^45^ ^]^ in moiré acoustic metasurfaces and later verified by Han et al^[^ ^46^ ^]^ through both experimental and theoretical work, extending the concept to multilayer acoustic metasurfaces.

To further expand the utility of bilayer metasurfaces and their rich physics, in this work, we explore the layer homogeneity degree‐of‐freedom and study its impact on wave manipulation. Unlike traditional moiré metasurfaces that utilize a single lattice type, we constructed our moiré metasurface using two distinct lattices in the individual layers: one being a square lattice and the other a triangular lattice. By precisely controlling the coupling between the two different lattice layers, we can modulate the dispersion characteristics of the moiré metasurface, achieving a topological transition from the hyperbolic regime to the elliptical regime. During this process, there exists a specific twist angle where the dispersion curve flattens, offering the potential for low‐loss^[^ ^47^, ^48^ ^]^sound wave propagation. Moreover, the excellent robustness of the proposed moiré metasurface was demonstrated by introducing line defects into the tHB system, where results from theoretical derivation, numerical simulations, and experimental validation all confirmed this phenomenon. This work not only provides new insights into topological phase transitions in acoustics but also offers greater flexibility in controlling the interaction between sound waves and medium, potentially laying the foundation for the design and application of future acoustic devices.

## 2 Results and Discussion

**Figure 1:**
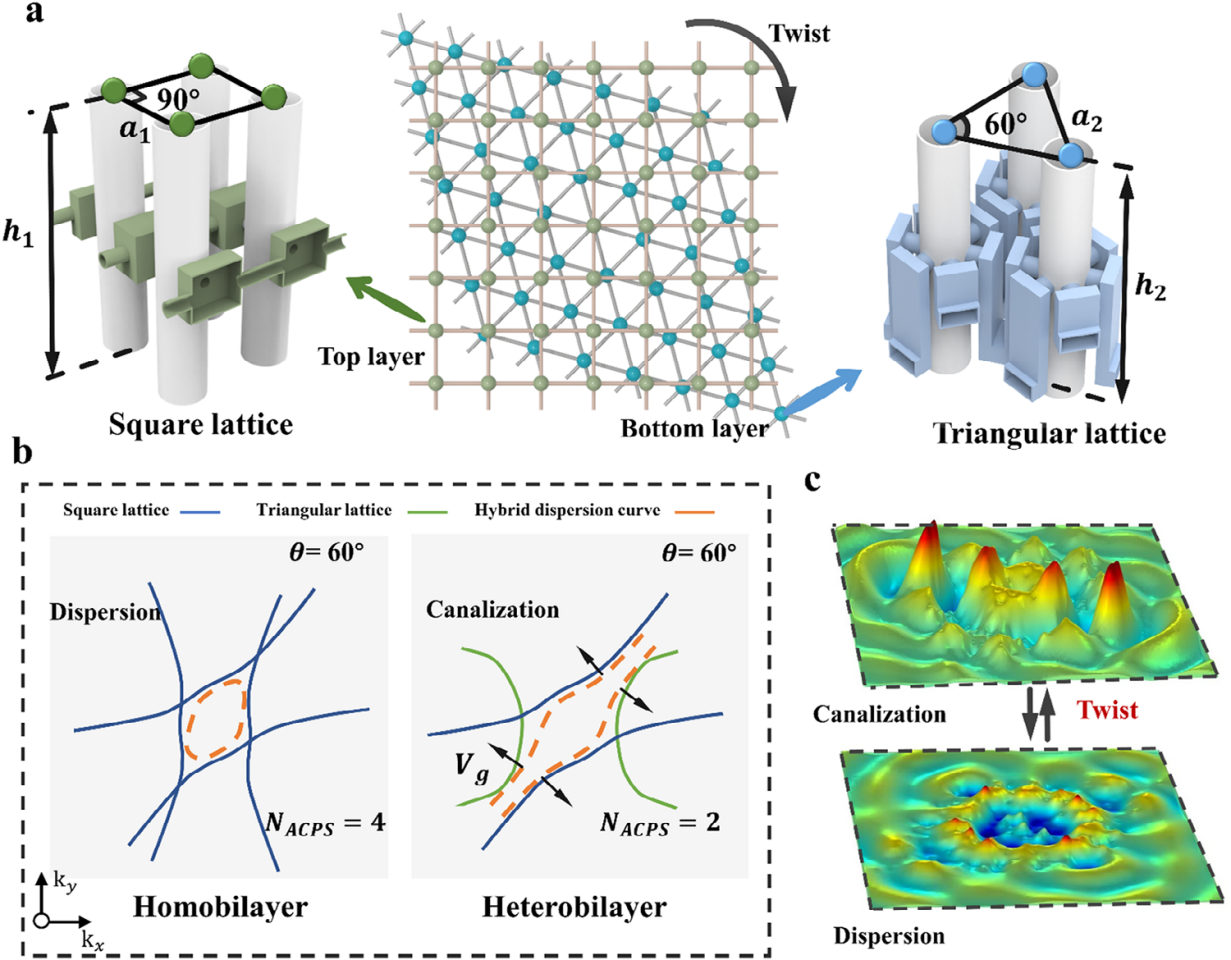
Hyperbolic surface polarization of the heterobilayer system. a) Schematic diagram of the square and triangular lattices, with a conceptual illustration of the heterobilayer structure; green represents the square lattice, and blue represents the triangular lattice. b) The blue solid line and green solid line represent the dispersion curves of the square and triangular lattices, respectively, while the orange dashed line represents the hybridized dispersion curve of both lattices. The black arrow indicates the direction of the group velocity. This illustrates the hybridization of the dispersion curves in homogeneous and heterogeneous bilayer structures at a twist angle of 60°. At this angle, the homogeneous bilayer exhibits an elliptical topological state, while the heterogeneous bilayer shows a hyperbolic topological state. c) 3D visualization of sound field patterns between hyperbolic and elliptical modes.

The schematic of the proposed tHB system is illustrated in **Figure** **1**a, which differs from traditional moiré bilayer acoustic metasurfaces as it comprises two layers of different lattices. The upper layer consists of a square lattice with a unit cell side length of *a*_1_ = 42 mm and a height of *h*_1_ = 110 mm. The lower layer features a triangular lattice, characterized by a unit cell side length of *a*_2_ = 40 mm and a height of *h*_2_ = 100 mm. The specific parameters are provided in Section S1 (Supporting Information). The distance between the two layers is 0.1*a*_2_, and the coupling between the two layers is typically achieved through an air gap. In this configuration, the air domain acts as an intermediary, allowing sound waves to propagate and interact between the layers. Notably, as the coupling distance increases, the coupling strength gradually weakens, and when the distance exceeds *a*_2_, the coupling is almost completely decoupled.^[^ ^45^ ^]^ We measured the sound pressure transmission rate of the tHB system at different interlayer coupling distances (0.3*a*_2_, 0.5*a*_2_, *a*_2_, 2*a*_2_, 3*a*_2_), as shown in Figure S7 (Supporting Information). When the interlayer distance is greater than *a*_2_, the phenomenon becomes less pronounced, and the sound pressure transmission rate decreases more gradually. The central illustration in Figure 1a presents the lattice configuration of the tHB system. To achieve extreme anisotropy, we employed a non‐local connection method to link each unit in the two lattices, thereby opening energy channels between adjacent unit cells and enabling hyperbolic propagation within the plane. The realization of hyperbolic propagation also requires that the imaginary parts of the coupling tube impedances in the different directions of the unit cell have opposite signs.

In photonic and elastic wave systems, the study of polaritons has been extensively explored. Hu et al^[^ ^49^ ^]^ explored how to control and manipulate the photonic dispersion of polaritons in van der Waals bilayers with extreme anisotropy. Yves et al^[^ ^50^ ^]^ investigated the hyperbolic shear polaritons on elastic wave metasurfaces in twisted‐induced crystals. Compared to the polaritons observed in photonic systems and elastic waves, the propagation of sound waves in acoustic systems exhibits similar polarization phenomena. In all these systems (i.e., photonic systems, elastic waves, and acoustic waves), when the external excitation frequency matches the characteristic frequency corresponding to the hyperbolic dispersion curve of their own, hyperbolic propagation is observed. Hu et al achieved the topological transition of the dispersion curve from *N_ACP_* = 2 to *N_ACP_* = 4 through the twisting angle between the bilayers, and the phonons we study also exhibit a similar topological transition. In the excitation frequency range between *f* = 1840 Hz and *f* = 2020 Hz, the adjustment of the twist angle between the two layers leads to a topological transition from hyperbolic to elliptical modes. This phenomenon arises from the hybridization of the dispersion curves of two distinct single layers, where the rotation of these curves collectively influences the overall dispersion curve of the tHB system. For detailed information on the dispersion curves, refer to Section S3 (Supporting Information).

This topological transition is related to a topological invariant, defined by the number of *N_ACPS_* in the heterogeneous bilayer metasurface. For a specific structure, the value of *N_ACPS_* is dependent on the rotational angle between the two layers and the magnitude of the excitation frequency. The excitation frequency influences the opening angle of the dispersion curve. As the excitation frequency increases, the opening angle of the dispersion curve gradually becomes smaller, thereby affecting the hybridization between the bilayer dispersion curves. Figure S4 (Supporting Information) illustrates the opening angle of two lattice dispersion curves at a frequency of *f* = 1880 Hz. Regarding the rotation angle, *N_ACPS_* undergoes a stepwise change by continuously modulating the twist angle, which serves as a measure to distinguish the hyperbolic mode and the elliptical mode. For example, when *N_ACPS_* = 2, the metasurface exhibits a hyperbolic mode, while when *N_ACPS_* = 4, it transitions to an elliptical mode.

The left and right sides of Figure 1b show the hybridization process of the dispersion curves in the homogeneous and heterogeneous bilayer structures at a relative twist angle of 60°. The blue solid line represents the dispersion curve of the square lattice, the green solid line represents the dispersion curve of the triangular lattice, and the orange dashed line represents the hybridized result of the blue and green dispersion curves. Within a specific frequency range, although both lattice types exhibit hyperbolic dispersion, their degree of hyperbolicity differs due to the structural differences between the lattices. This difference in hyperbolicity allows the tHB system to more flexibly regulate topological transitions, providing greater freedom in adjusting the dispersion characteristics. Specifically, in the homogeneous bilayer structure, when the twist angle is 60°, the hybridized dispersion curve (orange dashed line) closes, corresponding to *N_ACP_* = 4. In the heterogeneous bilayer structure, however, even at the same 60° twist angle, the dispersion curve remains open, corresponding to *N_ACP_* = 2, due to the differing lattice types in the two layers. This indicates that, compared to the homogeneous bilayer system, the tHB system has a significant advantage in controlling topological transitions and dispersion characteristics by flexibly combining different dispersion curves. As shown in Figure 1b, the tHB system and the homogeneous bilayer system can achieve completely different topological modes at the same twisting angle, significantly expanding their application flexibility in acoustic wave manipulation. By adjusting the topological characteristics, acoustic waves can be focused or scattered in specific directions, enabling high‐precision acoustic imaging or signal transmission, and facilitating beamforming and acoustic focusing.

Figure S3 (Supporting Information) illustrates the sound pressure propagation in the tHB system at different twisting angles, along with the corresponding 2D fast Fourier transform (FFT) of the sound pressure field. Figure 1c shows the 3D visualization of the topological transition in the sound field pattern, Video S1 (Supporting Information) demonstrates the process of 3D transformation through an animation. Notably, in the intermediate state between the hyperbolic and elliptical modes, there exists a nearly flat dispersion curve (see Figure S3b, Supporting Information). Unlike the conventional flat bands,^[^ ^51^, ^52^ ^]^ the flat bands in this study are induced by interlayer mode coupling, originating from the interaction between the dispersion curves of the two layers, resulting in flat regions. This type of flat band is dependent on the strength of interlayer coupling and the twist angle,^[^ ^45^, ^49^ ^]^ making it a unique phenomenon in bilayer or multilayer systems. Under acoustic flat‐band conditions, sound waves are strongly confined and exhibit minimal diffraction, showcasing exceptional transmission performance. When the rotation angle between the layers is 26°, the hybridized dispersion curve appears almost parallel, with the group velocity direction perpendicular to the dispersion curve. This results in minimal diffraction of the sound waves, and we refer to this angle as the “acoustic magic angle” in our tHB system. Due to the varying angles of the dispersion curves at different frequencies, the magic angle also changes accordingly. For a detailed illustration, please refer to Figure S5 (Supporting Information).

Figure S6 (Supporting Information) shows the energy distribution along the sound pressure propagation direction at various twisting angles. We found that as the twisting angle increases from 0°, the energy transmission efficiency gradually increases, reaching its peak when the twisting angle reaches the magic angle of 26°. This is because, at the magic angle, the dispersion curve hybridizes and flattens, enabling low‐loss propagation. When the twist angle exceeds 26°, the hybridized dispersion curves close, forming elliptical characteristics; below 26°, the dispersion curves intersect, clearly revealing the characteristics of the magic angle. As the twisting angle continues to increase, energy transmission gradually decreases. In contrast, at a twisting angle of 90°, the hybridized dispersion curve closes, resulting in elliptical propagation. In this case, energy propagation lacks directionality, causing the energy transmission efficiency to drop to its lowest point. Moreover, the dispersion curve of the tHB system results from the hybridization effect between two different dispersion curves. As a result, hybridization can help to achieve different magic angle values, which is not possible using homogeneous bilayer metasurfaces. In practical applications, heterogeneous bilayer metasurfaces offer more flexible designs for sound beam deflection and focusing. Different magic angles can correspond to wave low‐loss propagation in different directions, greatly enhancing application versatility. By adjusting the rotation angle, precise control over sound wave propagation direction can be achieved, making it suitable for acoustic communication and other fields.

**Figure 2:**
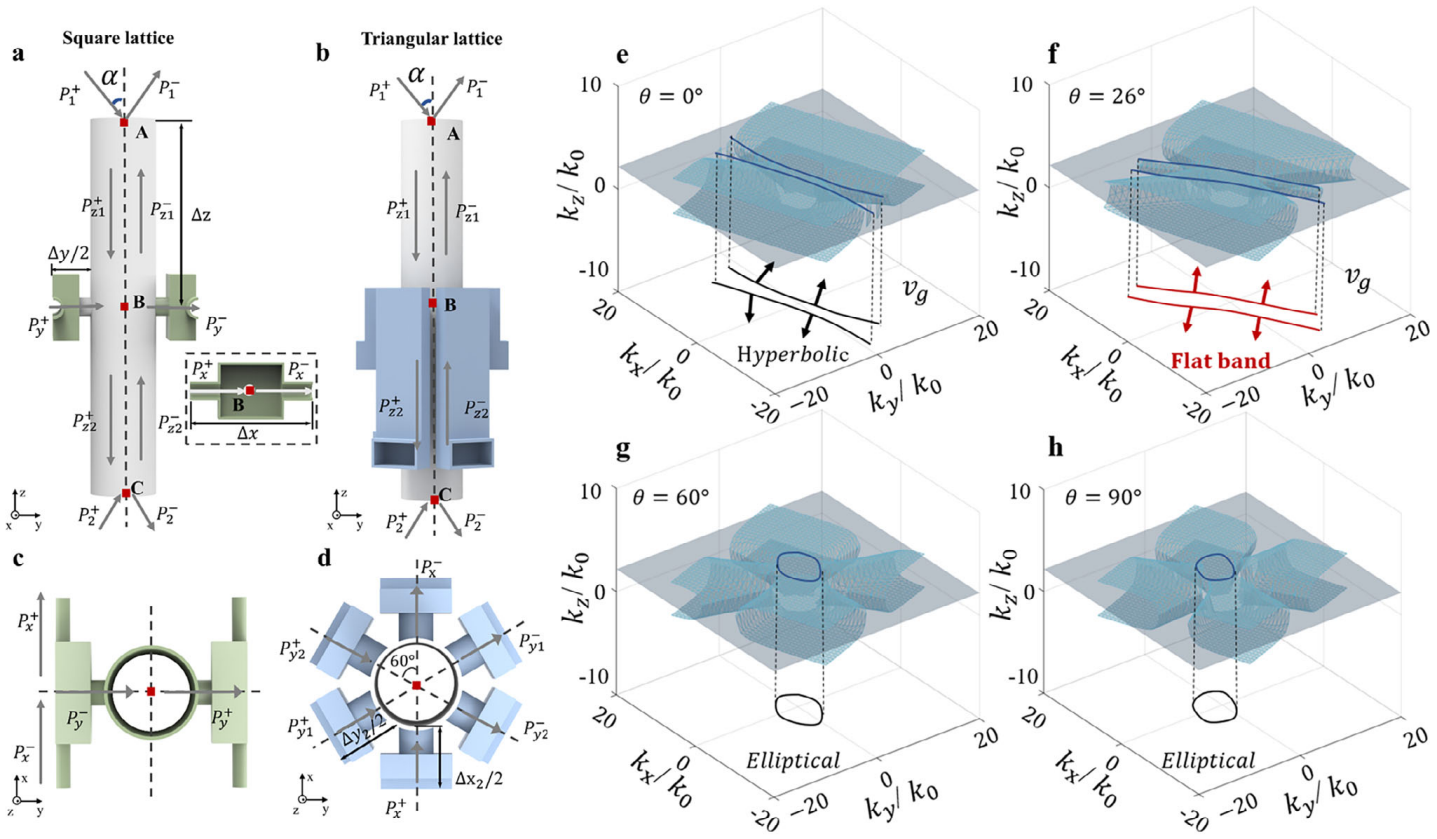
Theoretical model of the tHB system. a, c) The front and top views of the square unit cell, respectively. b, d) The front and top views of the triangular unit cell. P_1_ and P_2_ are the input and output acoustic pressures. Δx and Δy represent the lengths of the coupled tubes and 𝛼 represents the incident angle. e) Hyperbolic propagation in the tHB system without twisting. f) Flat band formation resulting from a 26° twist (acoustic magic angle). g, h) Elliptical propagation generated by twisting the tHB system at angles of 60° and 90°. *k_z_* is the z‐component of the wavevector in air. *k*_0_ refers to the magnitude of the wavevector in free space.

To gain a deeper understanding of the characteristics of the heterobilayer moiré system, we first present the theoretical framework to clarify the mechanism of the hybridized dispersion. **Figure** **2**a,c show the front and top views of the square unit cell, respectively, while Figure 2b,d illustrate the front and top views of the triangular unit cell. By applying the Bloch‐Floquet theorem, we can resolve the dispersion characteristics of a monolayer metasurface, the relationship between acoustic waves is described by (refer to Section S2, Supporting Information).

(1) $$\left(\begin{array}{c} P_{1}^{+}\\ P_{1}^{-} \end{array}\right)=\left(\begin{array}{cc} H_{11} & H_{12}\\ H_{21} & H_{22} \end{array}\right)\;\left(\begin{array}{c} e^{-ik_{z}d_{h}}P_{1}^{+}\\ e^{-ik_{z}d_{h}}P_{1}^{-} \end{array}\right)$$

where *P*_1_ and *P*_2_ are the input and output acoustic pressures. *d_h_* is the height of the z‐direction resonator (points A to C). ***H***_11_, ***H***_12_, ***H***_21_, ***H***_22_ represent the matrix elements describing the relationship between the sound pressures $P_{1}^{+}$ and $P_{1}^{-}$. *k_z_* is the z‐component of the wavevector in air. To ensure the solution of this matrix equation, the following relation needs to be satisfied:

(2) $$\left|\begin{array}{cc} H_{11}-e^{ik_{z}d_{h}} & H_{12}\\ H_{21} & H_{22}-e^{ik_{z}d_{h}} \end{array}\right|=0$$

We consider the top and bottom metasurfaces to be composed of triangular and square lattices, respectively. Compared to the square lattice, the triangular lattice includes six coupling channels, enabling the conversion of continuous sound pressure and mass conservation in various directions into the x and y directions:

(3) $$U_{y}^{-}=sin\left(60^{\circ }\right)U_{y1}^{-}+sin\left(60^{\circ }\right)U_{y2}^{-}$$

(4) $$U_{x}^{-}=U_{x}^{-}+cos\left(60^{\circ }\right)U_{y2}^{-}-\cos\left(60^{\circ }\right)U_{y2}^{+}$$

$U_{x}^{-}$ and $U_{x}^{+}$ correspond to the outflow and inflow volume velocities in the x‐direction, respectively. $U_{y}^{+}$ and $U_{y}^{-}$ represent the volume velocities from the y‐direction. This model is further expanded to encompass heterobilayer systems by employing a transfer matrix approach:

(5) $$H_{1\to 3}=H_{1\to 2}\;H_{d_{1}}H_{2\to 3}$$

with

(6) $$H_{d_{1}}=\left(\begin{array}{cc} e^{ik_{z}d_{1}} & 0\\ 0 & e^{-ik_{z}d_{1}} \end{array}\right)\;$$

where the method for obtaining ${\hat{T}}_{2\to 3}$ is similar to that of getting ${\hat{T}}_{1\to 2}$. ${\hat{T}}_{d_{1}}$ is the transfer matrix of the wave propagating through the interlayers with thickness *d_j_*.

The theoretical model is also capable of predicting the dispersion response of the tHB system, as illustrated in Figure 2e–h. When the relative rotation angle between the two layers of the metasurface is 0° (see Figure 2e), the dispersion curve of the tHB system exhibits a typical hyperbolic shape, indicating that the system displays anisotropic characteristics, with strongly directional sound wave propagation. However, as the relative rotation angle between the layers increases to 60° and 90° (see Figure 2g,h), the dispersion curve gradually contacts and closes, resulting in a more uniform wave propagation characteristic. In this condition, the direction of sound wave propagation becomes less dependent on specific orientations, showcasing isotropic propagation properties. This phenomenon indicates that by varying the rotation angle, the acoustic characteristics of the tHB system can transition from anisotropic to isotropic, thereby enabling a broader range of tunability.

Notably, when the rotation angle between the two layers reaches 26° (see Figure 2f), the dispersion curve features an exceptionally flat profile indicated by the red line. In this scenario, the direction of the group velocity is represented by the red arrow in the figure, which is perpendicular to the tangent of the dispersion curve. At this angle, sound wave propagation becomes highly directional and collimated, exhibiting remarkable directionality immune to diffractions. This unique dispersion characteristic is confirmed by numerical simulation results shown in Figure S3b (Supporting Information) and endows the tHB system with tremendous potential in the field of acoustic manipulation. The flat dispersion curve will enable efficient sound wave transmission control in practical applications where highly directional energy concentration is desired.

**Figure 3:**
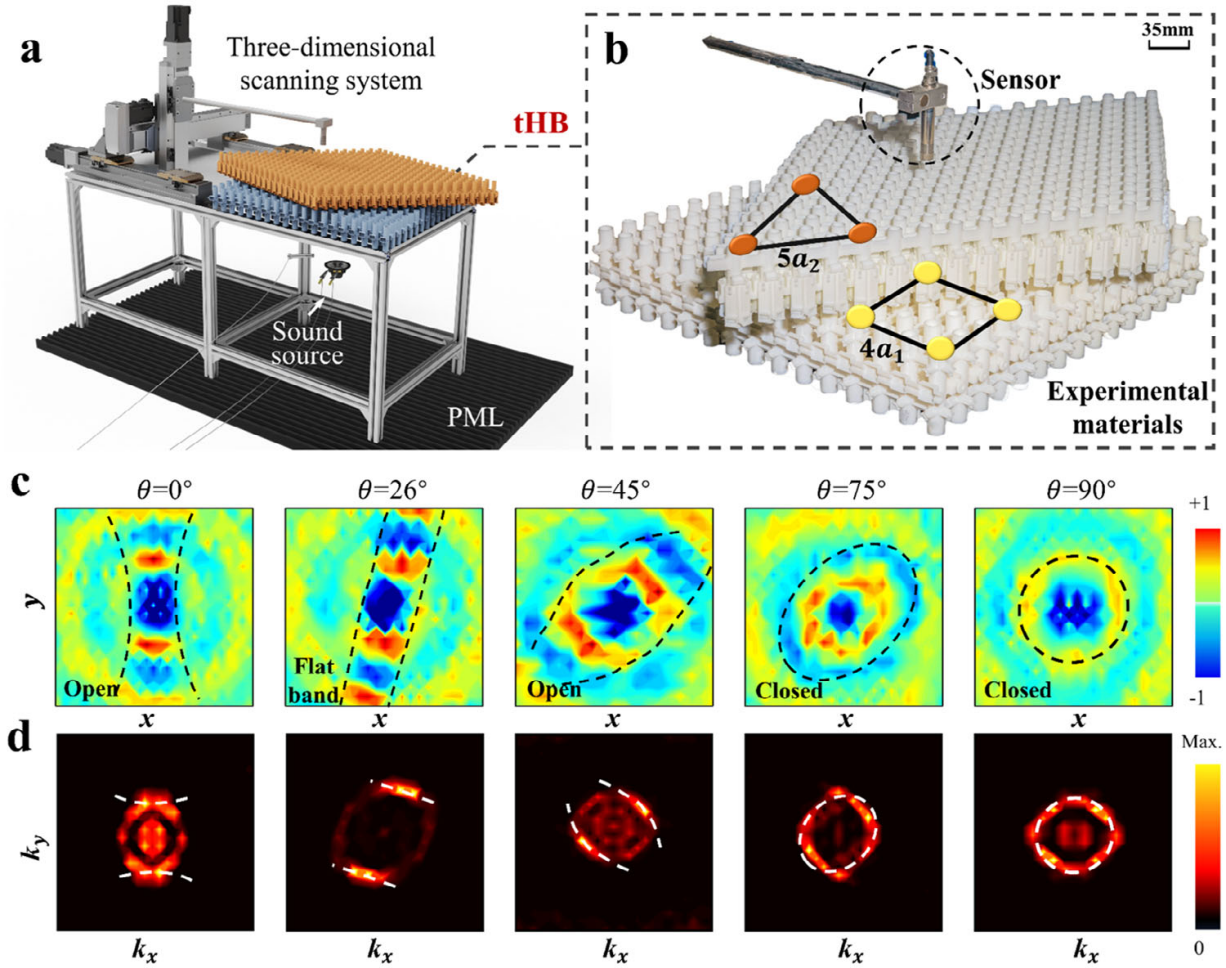
Experimental observations of the tHB system at a frequency of *f* = 1880 Hz. a) Schematic diagram of the acoustic experimental setup used to measure the tHB metasurface, including the sound source, PML, tHB, sensor, and the 3D scanning system. b) 3D‐printed physical experimental materials in the tHB system, the orange triangle represents a length of 5 lattice units of a_1_ while the blue square represents 4 lattice units of a_2_. c) Experimental measurements of sound field distribution in the tHB system at 0°, 26°, 45°, 75°, and 90°, the black dashed line is the guiding line for sound pressure propagation. d) The corresponding FFT transformation of the experimentally obtained sound fields, with the white dashed line representing the hybridized dispersion curve of the tHB system at different twisting angles.

Following the theoretical analysis and numerical simulations, we further validated the effectiveness and reliability of the simulation results through experimental methods. In the experiment, a small sound source was placed beneath the tHB metasurface to simulate a point source, with a frequency of *f* = 1880 Hz, as illustrated in **Figure** **3**a. An acoustic probe is placed 10 mm above the metasurface and is controlled to move within the 2D space to collect sound pressure distribution data. Another acoustic probe was fixed beneath the metasurface to obtain reference sound pressure data. The perfectly matched layer (PML) is placed beneath the metasurface to absorb sound. To minimize interference from reflected waves in the experimental environment, the metasurface is placed in an open space, ensuring a low reflection surrounding. As shown in Figure 3b, we utilized high‐precision 3D printing technology to fabricate the tHB system composed of two distinct lattices. It is important to note that the support material in 3D printing may affect the structure of the air cavity. To mitigate this, we adopted a modular approach by printing individual units and assembling them. Additionally, we increased the surface area at the bonding sites of each unit to enhance adhesion and ensure a more secure connection.

We measured the sound wave propagation behavior under various moiré bilayer angle conditions, namely at angles of 0°, 26°, 45°, 75°, and 90° which encompass a full range covering the transition between different states. The processed sound wave propagation field obtained from the experimental sound pressure data is presented in Figure 3c. The black dashed line in the figure indicates the guiding line for sound pressure propagation. This clearly illustrates the experimentally measured sound fields at different angles, revealing the transition from hyperbolic propagation in the open‐state dispersion to elliptical propagation in the closed‐state dispersion. Furthermore, to gain a comprehensive understanding of the characteristics of sound wave propagation, we performed an FFT on the sound pressure data, yielding the corresponding Fourier spectrum in the momentum space as illustrated in Figure 3d. The white dashed line marks the hybridized dispersion curves at different twist angles. Meanwhile, the brightness distribution of the FFT clearly shows the dispersion characteristics in reciprocal space. We observed that the hybridized dispersion curves align closely with the regions of brightness variation in the FFT, which further proves the accuracy of the experimental measurements and the reliability of the data analysis. By comparing this with the numerical simulation results, we found a high degree of consistency between the experimental Fourier spectrum and the simulation results shown in Figure S3 (Supporting Information). This agreement indicates that the experiment not only successfully validated the accuracy of the experiment results but also further substantiated our understanding of the acoustic behavior of the tHB system. In summary, this experiment not only confirmed the reliability of the simulation model and theoretical predictions of the tHB system but also demonstrated the system's excellent performance and operability in acoustic manipulation.

**Figure 4:**
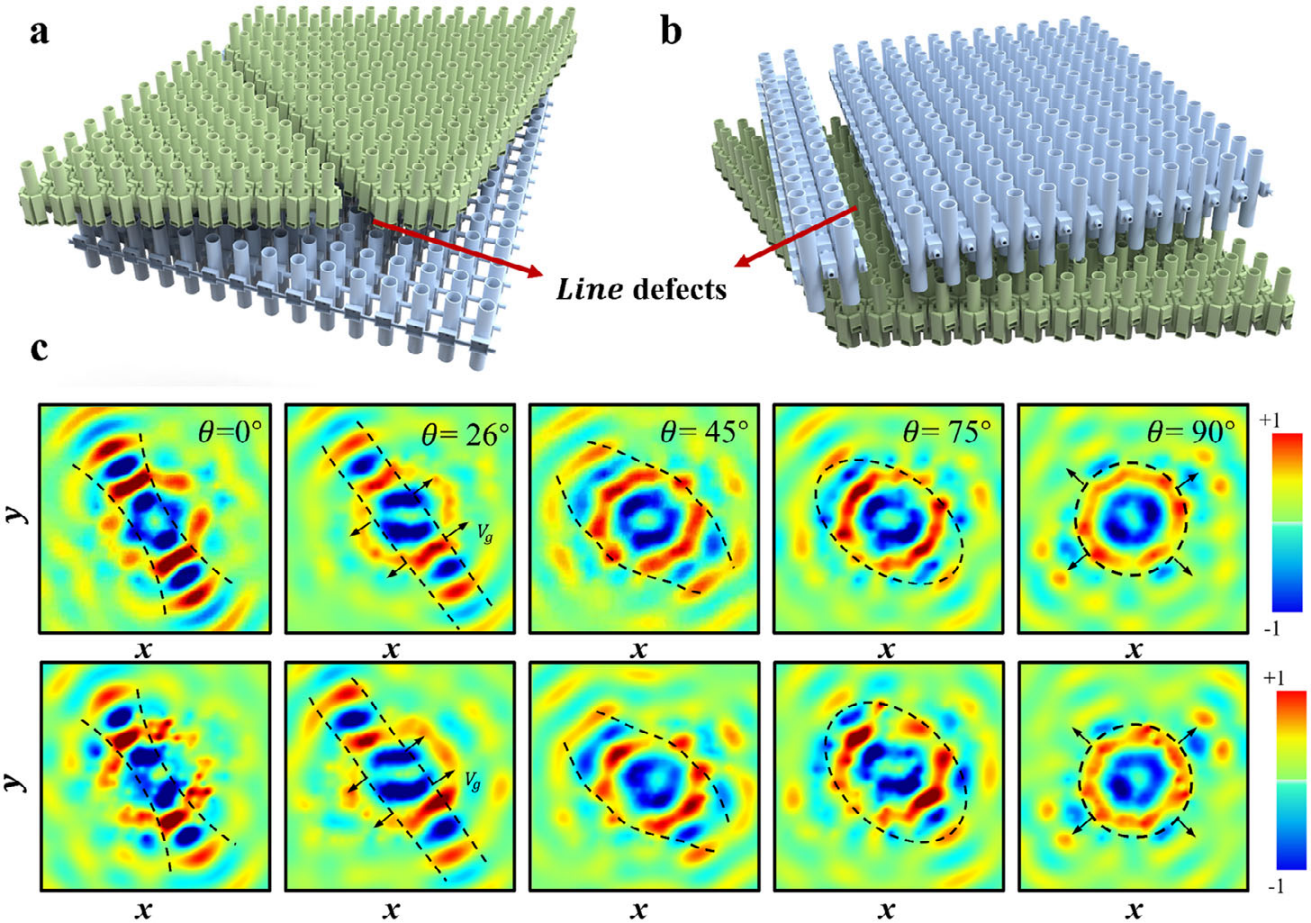
Robustness of wave propagation to line defects. a) Schematic representation of line defects in the triangular lattice layer of the tHB system. b) Schematic representation of line defects in the square lattice layer of the tHB system. c) Pressure field of the tHB system with line defects at various angles at a frequency of *f* = 1880 Hz, the black dashed lines represent the dispersion curves, and the arrows indicate the direction of group velocity. The simulated fields closely resemble the ones without defects, demonstrating the robustness of the system.

It is noteworthy that the tHB system exhibits remarkable robustness, enabling sound waves to propagate with the existence of defects or irregularities without affecting their energy and directionality. Such characteristics are crucial for maintaining the stability of acoustic systems in real‐world applications. Traditional sound wave systems are often susceptible to the impact of minor defects in materials or structures, which can lead to performance degradation. Robust wave propagation with minimal distortion to imperfections, on the other hand, can greatly enhance their integrity and operability. In our experiments, despite the possible dimensional errors stemming from the small features of micro‐scale lattice structures, the experimental results (see Figure 3) closely aligned with the simulation results, providing substantial evidence of the system's robustness and indicating the feasibility and stability of the tHB system in practical manufacturing and applications. To further validate this, we conducted a simulation analysis of line defects in the different lattice layers. **Figure** **4**a shows the line defects in the triangular lattice, while Figure 4b presents those in the square lattice. Keeping other simulation conditions unchanged, Figure 4c illustrates that the sound pressure propagation path is nearly unaffected by these line defects.

Additionally, in the tHB system, sound waves are able to automatically circumvent structural defects during propagation, avoiding reflection or attenuation caused by localized structural damage or irregularities. This phenomenon is a direct manifestation of topological protection, which allows sound waves to propagate uninterruptedly along protected boundaries or interfaces. This powerful robustness has profound implications for numerous practical applications. Particularly in acoustic applications that require high precision and stability, such as non‐destructive sensing, precise sound wave control, and ultrasonic imaging, the tHB system is expected to significantly enhance the performance of devices. Furthermore, it opens up vast possibilities for the design of novel acoustic devices. As acoustic devices evolve toward higher precision and more complex structures, overcoming uncertainties such as structural defects and material errors in practical manufacturing becomes a key challenge. The tHB system, due to its topological protection characteristics, can maintain exceptional performance even under these constraints, significantly improving the device's resistance to interference and reliability.

## 3 Conclusion

This study provides an in‐depth exploration of the applications of the tHB system in acoustic metasurfaces. The tHB system achieves extreme anisotropy through non‐local connections between two different lattices in the upper and lower layers, facilitating a topological transition between hyperbolic and elliptical modes that is related to the number of *N_ACPS_* by adjusting the interlayer angle. Compared to homogeneous bilayers, they provide greater angular control, resulting in more flexible hybridization of the dispersion curves. Notably, we discovered that at a specific twisting “magic angle”, the dispersion curve hybridizes into a flat band, demonstrating exceptional transmission performance. We also confirmed the system's dispersion response and sound pressure propagation characteristics, demonstrating its remarkable robustness against line defects and irregularities. The experimental results align closely with the simulations, further indicating the tHB system's excellent operability and application potential. The highly controllable wave propagation direction and strong collimation characters are especially useful in acoustic applications such as non‐destructive sensing and precise acoustic control, where highly directional wave control is needed. Finally, we anticipate that the differences in dispersion curves introduced by heterogeneous bilayers will provide moiré metasurfaces with a broader frequency response range and more adaptive wave mode selection, enhancing their capability for controlling wave propagation. It is envisioned that with the new design degree‐of‐freedom offered by the proposed tHB system, other unique wave effects may also be realized through the interplay between heterobilayer structures.

## Conflict of Interest

The authors declare no conflict of interest.

## Author Contributions

S.‐D.F. and C.‐L.H. contributed equally to this work. T.‐Z.Y., C.S., H.‐T.S., and L.‐Q.C. conceived the idea. S.‐D.F., C.‐L.H., and H.K. performed the experiments. S.‐D.F., C.‐L.H., H.K., and L.B. carried out the theoretical analysis and simulations. S.‐D.F., C.S., and T.‐Z.Y. wrote the manuscript with contributions from the other authors. All of the authors have made a substantial contribution to the paper.

## Supporting information



Supporting Information

Supplemental Video 1

## Data Availability

The data that support the findings of this study are available from the corresponding author upon reasonable request.
