# Supplementary material for: Acoustic Moiré Flat Bands in Twisted Heterobilayer Metasurface
Source: Adv Mater. 2025 May 9;37(29):2418839. doi: 10.1002/adma.202418839 (PMC12288825; doi:10.1002/adma.202418839)
Supplement: Supplementary file 1 — Supporting Information [file ADMA-37-2418839-s001.docx]

**Supplementary Information**

**Acoustic moiré flat bands in twisted heterobilayer metasurface**

Shida Fan^1^, Chenglin Han^1^, Kuan He^1^, Liang Bai^1^, Li-Qun Chen^2^, Huaitao Shi^3^*, Chen Shen^4^*, Tianzhi Yang^1^*

*^1^School of Mechanical Engineering and Automation, Northeastern University, 110819 Shenyang, China*

*^2^School of Science, Harbin Institute of Technology, 518055 Shenzhen, China*

*^3^School of Mechanical Engineering, Shenyang Jianzhu University, 110168 Shenyang, China*

*^4^Department of Mechanical Engineering, Rowan University, Glassboro, New Jersey 08028, USA*

**Corresponding authors:** Prof. Huaitao Shi, [sht@sjzu.edu.cn](mailto:sht@sjzu.edu.cn)

Prof. Chen Shen, shenc@rowan.edu

Prof. Tianzhi Yang, yangtianzhi@me.neu.edu.cn

Section 1. Details of the unit cell structure parameters.

The heterogeneous bilayer moiré metasurface is composed of a square lattice paired with a triangular lattice., connected non-locally. We have designed two structures: the square lattice has a lattice constant *a*_1_ = 42 mm, and the specific parameters are shown in Figure S1a. The coupling tubes are used to connect the main tubes in two directions, the advantage of this design is that it provides greater structural flexibility. The triangular lattice has a lattice constant *a*_2_ = 40 mm, with specific parameters shown in Figure S1b. It features six directions of coupling tubes, including four identical long coupling tubes and two identical short coupling tubes. The differing lengths of these tubes enable the structure to achieve extreme anisotropy, as they satisfy the property of opposite signs for the imaginary part of the acoustic impedance at specific frequencies.


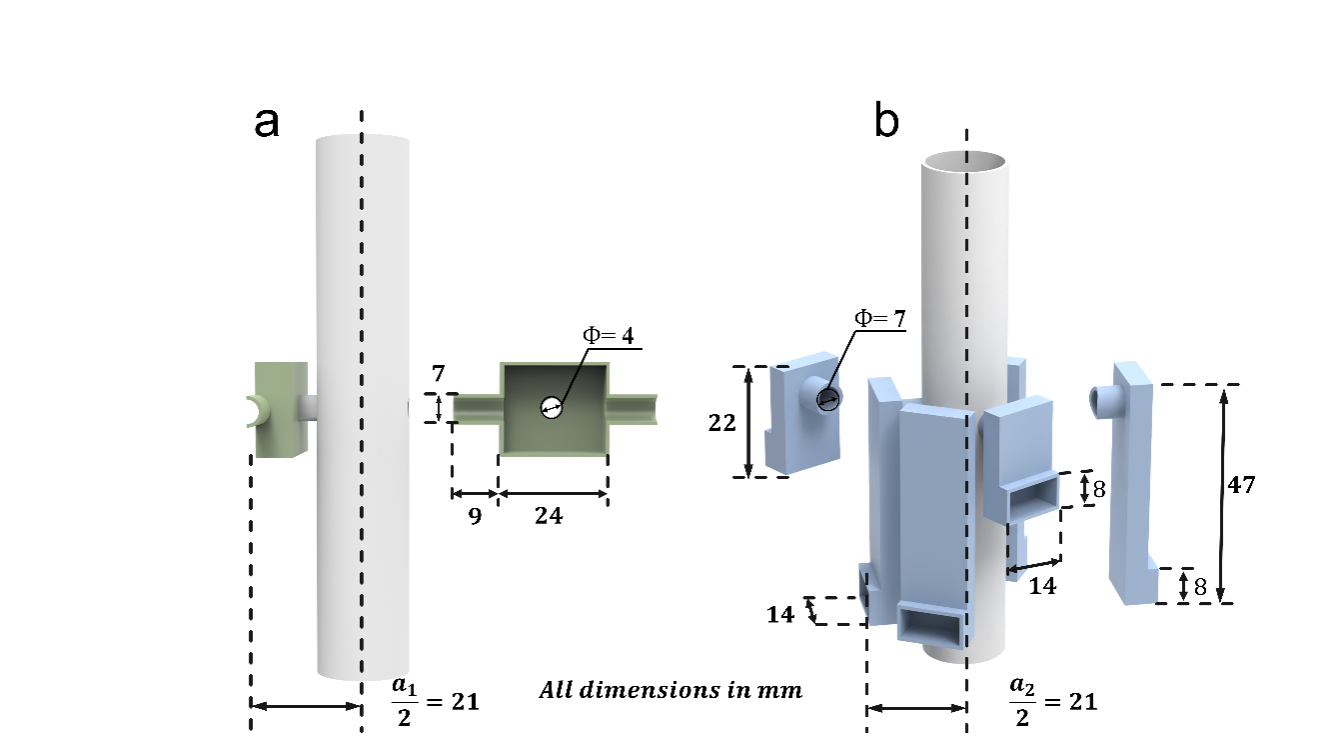


**Figure S1.** The unit cell parameters of the two structures in the heterogeneous bilayer. a) the parameters of the square lattice structure. b) the parameters of the triangular lattice structure.

Section 2. Derivation of dispersion relations

Firstly, we derive the dispersion relation of the square lattice. As shown in Figure 2a, the normal mass velocity and sound pressure continuity at point A are required:

| $-\frac{k_{z}}{k_{0}\rho c}P_{1}^{+}+\frac{k_{z}}{k_{0}\rho c}P_{1}^{-}=-\frac{1}{\rho c}P_{z1}^{+}+\frac{1}{\rho c}P_{z1}^{-}$ | (S1) |
| --- | --- |
| $P_{1}^{+}+P_{1}^{-}=P_{z1}^{+}+P_{z1}^{-}$ | (S2) |

Here, $c$ is the sound speed in air, $\rho$ is the air density, $k_{0}=\frac{\omega}{c}$ and$\cos(\alpha)=\frac{k_{z}}{k_{0}}$. $P_{1}$ and $P_{2}$ are the input and output acoustic pressures. $k_{z}$ is the z-component of the wavevector in air. $k_{0}$ refers to the magnitude of the wavevector in free space.

Eq. (S1) and (S2) are formulated with point A as the reference, where the z-axis aligns with the black vertical dashed line in Figure 2a. This reference point is chosen irrespective of the initial phase. Similarly, when point B is considered as the reference point, expressions are derived based on the continuity of sound pressure and mass conservation.

| $P\left( x,y,z \right)=P_{z1}^{-}+P_{z1}^{+}=P_{z2}^{+}+P_{z2}^{-}$ | (S3) |
| --- | --- |
| $U_{z2}^{+}-U_{z2}^{-}+U_{x}^{-}-U_{x}^{+}+U_{y}^{-}-U_{y}^{+}=U_{z1}^{+}-U_{z1}^{-}$ | (S4) |

At point B, $U_{z1}^{-}$ and $U_{z1}^{+}$ denote the volume velocities of outflow and inflow from the upper section of the tube in the z-direction, which $U_{z1}^{+}=\frac{S}{\rho c}P_{Z1}^{-}e^{-ik_{z}\Delta z}$,$U_{z1}^{-}=\frac{S}{\rho c}P_{Z1}^{+}e^{ik_{z}\Delta z}$. $U_{z2}^{-}$ and $U_{z2}^{+}$ represent the volume velocities of inflow and outflow in the lower half of the tube, which $U_{z2=}^{+}=\frac{S}{\rho c}P_{Z2}^{-}$ $U_{z2}^{-}=\frac{S}{\rho c}P_{Z2}^{+}$ .$U_{x}^{-}$ and $U_{x}^{+}$ correspond to the outflow and inflow volume velocities in the x-direction, which $U_{x}^{-}=U_{x}\left( x,y,z \right)$ ${U_{x}^{+}=U}_{x}\left( x-\Delta x,y,z \right)$. $U_{y}^{+}$ and $U_{y}^{-}$ represent the volume velocities from the y-direction and $U_{y}^{-}=U_{y}\left( x,y,z \right)$ $U_{y}^{+}=U_{y}\left( x,y-\Delta y,z \right)$. P(x,y,z) indicates the sound pressure at point B. Here, S represents the cross-sectional area of the z-direction resonator. Eq.(S4) can be specifically expressed as:

| $-\frac{S}{\rho c}P_{z1}^{+}e^{ik_{z}\Delta z}+\frac{S}{\rho c}P_{z1}^{-}e^{-ik_{z}\Delta z}=\frac{\partial U_{x}\left( x,y,z \right)}{\partial x}\Delta x+\frac{\partial U_{y}\left( x,y,z \right)}{\partial y}\Delta y-\frac{S}{\rho c}P_{z2}^{+}+\frac{S}{\rho c}P_{z2}^{-}$ | (S5) |
| --- | --- |

The acoustic impedances ($Z$) of the coupling tubes in the *x-* directions and *y*-directions can be expressed as $Z_{x}$ and $Z_{y}$, respectively. $U_{x}\left( x,y,z \right)=-\frac{\partial P\left( x,y,z \right)}{Z_{x}\left( x,y,z \right)\partial x}\Delta x$ $U_{y}\left( x,y,z \right)=-\frac{\partial P\left( x,y,z \right)}{Z_{y}\left( x,y,z \right)\partial y}\Delta y$. Here, $\Delta x$, $\Delta y$ and $\Delta z$ represent the distances in the *x*-, *y*- and *z*-directions, respectively, as shown in Figure 2. By substituting the expressions of $U_{z1}$, $U_{z2}$, $U_{x}$ and $U_{y}$ into Eq. (S5), we obtain:

| $-\frac{S}{\rho c}P_{z1}^{+}e^{ik_{z}\Delta z}+\frac{S}{\rho c}P_{z1}^{-}e^{-ik_{z}\Delta z}=\left( \frac{S}{\rho c}+\frac{k_{x}^{2}\Delta x^{2}}{Z_{x}\left( x,y,z \right)}+\frac{k_{y}^{2}\Delta y^{2}}{Z_{y}\left( x,y,z \right)} \right)P_{z2}^{-}+(-\frac{S}{\rho c}+\frac{k_{x}^{2}\Delta x^{2}}{Z_{x}\left( x,y,z \right)}+\frac{k_{y}^{2}\Delta y^{2}}{Z_{y}\left( x,y,z \right)})P_{Z2}^{+}$ | (S6) |
| --- | --- |

As shown in Figure 2b and d, the triangular lattice and the square lattice differ in the installation angle of the coupling tube in the x-and y-direction, which leads to the difference in the expression obtained by the continuity of sound pressure and the conservation of mass of the triangular lattice at point B from that of the square lattice. But the idea of the derivation is the same, we just need to bring $U_{y}^{-}={sin(60^{\circ})U}_{y1}^{-}+{sin(60^{\circ})U}_{y2}^{-}$, $U_{y}^{+}=sin(60^{\circ})U_{y1}^{+}+{sin(60^{\circ})U}_{y2}^{+}$, $U_{x}^{+}=U_{x}^{+}-cos\left( 60^{\circ} \right)U_{y1}^{-}+{\cos\left( 60^{\circ} \right)U}_{y1}^{+}$and $U_{x}^{-}=U_{x}^{-}+cos\left( 60^{\circ} \right)U_{y2}^{-}-{\cos\left( 60^{\circ} \right)U}_{y2}^{+}$in a triangular lattice into Eq. (S4), a new relation at point B is derived according to the same idea as Eq. (S4) to Eq. (S6):

| $-\frac{S}{\rho c}P_{z1}^{+}e^{ik_{z}\Delta z}+\frac{S}{\rho c}P_{z1}^{-}e^{-ik_{z}\Delta z}=\left( \frac{S}{\rho c}+\frac{k_{x}^{2}\Delta x^{2}}{Z_{x}\left( x,y,z \right)}+\frac{\sqrt{3}k_{y}^{2}\Delta y^{2}}{Z_{y}\left( x,y,z \right)} \right)P_{z2}^{-}+(-\frac{S}{\rho c}+\frac{k_{x}^{2}\Delta x^{2}}{Z_{x}\left( x,y,z \right)}+\frac{{\sqrt{3}k}_{y}^{2}\Delta y^{2}}{Z_{y}\left( x,y,z \right)})P_{Z2}^{+}$ | (S7) |
| --- | --- |

Notice that according to our structure, $U_{y1}^{-},U_{y1}^{+}, U_{y2}^{-}$ and $U_{y2}^{+}$ are equal. $Z_{y1}$ and $Z_{y2}$are also equal, and we are $Z_{y}$ as a unified symbol. The relationship at points A and B is the same as before. Then, repeat the following steps here to obtain the dispersion of the periodic structure of the triangular lattice.

Similarly, the continuity conditions for point C sound pressure and normal mass velocity require:

| $P_{z2}^{+}+P_{z2}^{-}=P_{2}^{+}+P_{2}^{-}$ | (S8) |
| --- | --- |
| $\frac{1}{\rho c}P_{z2}^{+}-\frac{1}{\rho c}P_{z2}^{-}=\frac{1}{\rho c}P_{2}^{+}-\frac{1}{\rho c}P_{2}^{-}$ | (S9) |

As the metasurface is positioned in a semi-infinite space, there are no reflections underneath ($P_{2}^{-}=0$). Using Eqs. (S1), (S2), (S3), (S6), (S8), and (S9), we can derive three transfer matrices. These matrices establish the relationship between the sound pressure components within the structure.

| $\left( \begin{aligned} &P_{1}^{+} \\ &P_{1}^{-} \end{aligned} \right)=\boldsymbol{H}_{A}\left( \begin{aligned} &P_{z1}^{+} \\ &P_{z1}^{-} \end{aligned} \right)$ | (S10) |
| --- | --- |
| $\left( \begin{aligned} &P_{z1}^{+} \\ &P_{z1}^{-} \end{aligned} \right)=\boldsymbol{H}_{B}\left( \begin{aligned} &P_{z2}^{+} \\ &P_{z2}^{-} \end{aligned} \right)$ | (S11) |
| $\left( \begin{aligned} &P_{z2}^{+} \\ &P_{z2}^{-} \end{aligned} \right)=\boldsymbol{H}_{C}\left( \begin{aligned} &P_{2}^{+} \\ &P_{2}^{-} \end{aligned} \right)$ | (S12) |

With

| $\boldsymbol{H}_{A}=\left( \begin{matrix} \frac{1}{2k_{Z}}+\frac{1}{2} & -\frac{1}{2k_{Z}}+\frac{1}{2} \\ -\frac{1}{2k_{Z}}+\frac{1}{2} & \frac{1}{2k_{Z}}+\frac{1}{2} \end{matrix} \right)$ | (S13) |
| --- | --- |
| $\boldsymbol{H}_{B}=\left( \begin{matrix} A^{*} & B^{*} \\ C^{*} & D^{*} \end{matrix} \right)$ | (S14) |
| $\boldsymbol{H}_{C}=\left( \begin{matrix} \frac{k_{0}+k_{z}}{2k_{0}} & 0 \\ \frac{k_{0}-k_{z}}{2k_{0}} & 0 \end{matrix} \right)$ | (S15) |
| $A^{*}=-\frac{\Delta x^{2}\rho c{k_{x}}^{2}e^{\left( \Delta zk_{z} \right)}}{SZ_{x}\left( e^{\left( 2\Delta zk_{z} \right)}+1 \right)}+\frac{SZ_{x}Z_{y}\left( e^{\left( \Delta zk_{z} \right)}+1 \right)-\Delta y^{2}\rho cZ_{x}{k_{y}}^{2}e^{\left( \Delta zk_{z} \right)}}{SZ_{x}Z_{y}\left( e^{\left( 2\Delta zk_{Z} \right)}+1 \right)}$ | (S16) |
| $B^{*}=-\frac{\Delta x^{2}\rho c{k_{x}}^{2}e^{\left( \Delta zk_{z} \right)}}{SZ_{x}\left( e^{\left( 2\Delta zk_{Z} \right)}+1 \right)}-\frac{SZ_{x}Z_{y}\left( e^{\left( \Delta zk_{z} \right)}-1 \right)+\Delta y^{2}\rho cZ_{x}{k_{y}}^{2}e^{\left( \Delta zk_{z} \right)}}{SZ_{x}Z_{y}\left( e^{\left( 2\Delta zk_{Z} \right)}+1 \right)}$ | (S17) |
| $C^{*}=\frac{\Delta x^{2}\rho c{k_{x}}^{2}e^{\left( \Delta zk_{z} \right)}}{SZ_{x}\left( e^{\left( 2\Delta zk_{z} \right)}+1 \right)}+\frac{SZ_{x}Z_{y}\left( e^{\left( \Delta zk_{z} \right)}-1 \right)+\Delta y^{2}\rho cZ_{x}{k_{y}}^{2}e^{\left( \Delta zk_{z} \right)}}{SZ_{x}Z_{y}\left( e^{\left( 2\Delta zk_{z} \right)}+1 \right)}$ | (S18) |
| $D^{*}=\frac{\Delta x^{2}\rho c{k_{x}}^{2}e^{(\Delta zk_{z})}}{SZ_{x}(e^{(2\Delta zk_{z})}+1)}+\frac{SZ_{x}Z_{y}(e^{(\Delta zk_{Z})}+1)+\Delta y^{2}\rho cZ_{x}{k_{y}}^{2}e^{(\Delta zk_{z})}}{SZ_{x}Z_{y}(e^{(2\Delta zk_{z})}+1)}$ | (S19) |

Specifically, three transfer matrices are derived with origins A, B, and C, respectively. These matrices are not standardized within a single coordinate system, meaning the phase is not taken into account. Therefore, to establish the relationship between the input and output of the sound wave, we need to associate them using the transfer matrix $\boldsymbol{H}_{d}$.

| $\left( \begin{aligned} &P_{1}^{+} \\ &P_{1}^{-} \end{aligned} \right)=\boldsymbol{H}_{1\to2}\left( \begin{aligned} &P_{2}^{+} \\ &P_{2}^{-} \end{aligned} \right)=\boldsymbol{H}_{A}\boldsymbol{H}_{d}\boldsymbol{H}_{B}\boldsymbol{H}_{d}\boldsymbol{H}_{C}\left( \begin{aligned} &P_{2}^{+} \\ &P_{2}^{-} \end{aligned} \right)$ | (S20) |
| --- | --- |

with

| $\boldsymbol{H}_{d}=\left( \begin{matrix} e^{ik_{z}d} & 0 \\ 0 & e^{-ik_{z}d} \end{matrix} \right)$ | (S21) |
| --- | --- |

where $d$ is the distance between two adjacent points. According to the Bloch-Floquet theorem:

| $\left( \begin{aligned} &P_{1}^{+} \\ &P_{1}^{-} \end{aligned} \right)=\boldsymbol{H}_{1\to2}\left( \begin{aligned} &P_{2}^{+} \\ &P_{2}^{-} \end{aligned} \right)=\left( \begin{matrix} \boldsymbol{H}_{11} & \boldsymbol{H}_{12} \\ \boldsymbol{H}_{21} & \boldsymbol{H}_{22} \end{matrix} \right)\left( \begin{aligned} &e^{-ik_{z}d_{h}}P_{1}^{+} \\ &e^{-ik_{z}d_{h}}P_{1}^{-} \end{aligned} \right)$ | (S22) |
| --- | --- |

where $d_{h}$ is the height of the z-direction resonator (points A to C). Because the equation has only one solution, we obtain:

| $\left\vert\begin{matrix} \boldsymbol{H}_{11}-e^{ik_{z}d_{h}} & \boldsymbol{H}_{12} \\ \boldsymbol{H}_{21} & \boldsymbol{H}_{22}-e^{ik_{z}d_{h}} \end{matrix} \right\vert=0$ | (S23) |
| --- | --- |

The dispersion relation for the monolayer metasurface is obtained by bringing the elements of $\boldsymbol{H}_{1\to2}$ calculated from Eq. (S20) into Eq. (S23).

The characterization of the twist effect is achieved by combining the wave vector of each layer with its corresponding twist angle $(\theta)$. A corresponding example of a monolayer twist is as follows:

| $\left( \begin{matrix} k_{x1} \\ k_{y1} \end{matrix} \right)=\left( \begin{matrix} \cos(\theta) & -\sin(\theta) \\ \sin(\theta) & \cos(\theta) \end{matrix} \right)\left( \begin{matrix} k_{x} \\ k_{y} \end{matrix} \right)$ | (S24) |
| --- | --- |

The twisted dispersion relation is obtained by replacing $k_{x}$ and $k_{y}$ in the dispersion relation equation with $k_{x1}$ and $k_{y1}$ in (Eq. (S24)).

Section 3. Dispersion curves of the tHB system

By applying periodic boundary conditions to different lattice structures and scanning their Brillouin zones using COMSOL Multiphysics, we were able to obtain the characteristic frequencies and plot the corresponding iso-frequency contours. As shown in Figure S2a, the iso-frequency diagram for the triangular lattice is presented, while Figure S2b displays the iso-frequency diagram for the square lattice. It is evident that both lattices exhibit hyperbolic dispersion curves at the same frequency. Moreover, when symmetry is broken, the dispersion curvess in both lattices can become deflected and distorted, which significantly affects the wave propagation characteristics. This distortion and deflection are also the reason why the two iso-frequency diagrams appear rotated by a certain angle. These changes highlight the role of symmetry-breaking in manipulating the direction and behavior of wave propagation, providing valuable insights for designing materials with tunable waveguiding properties.


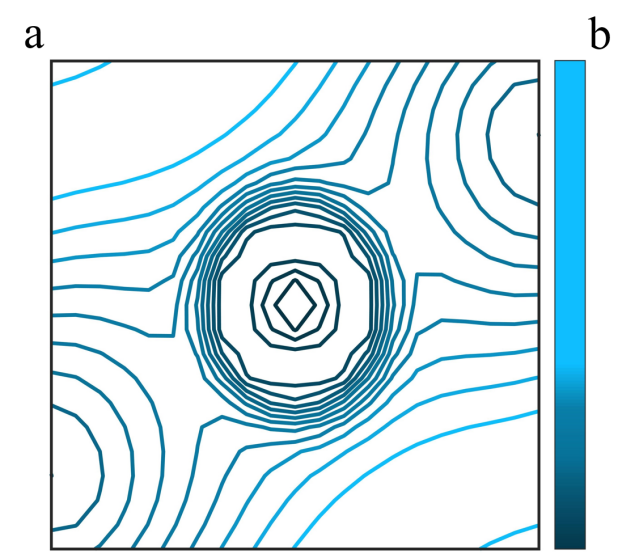

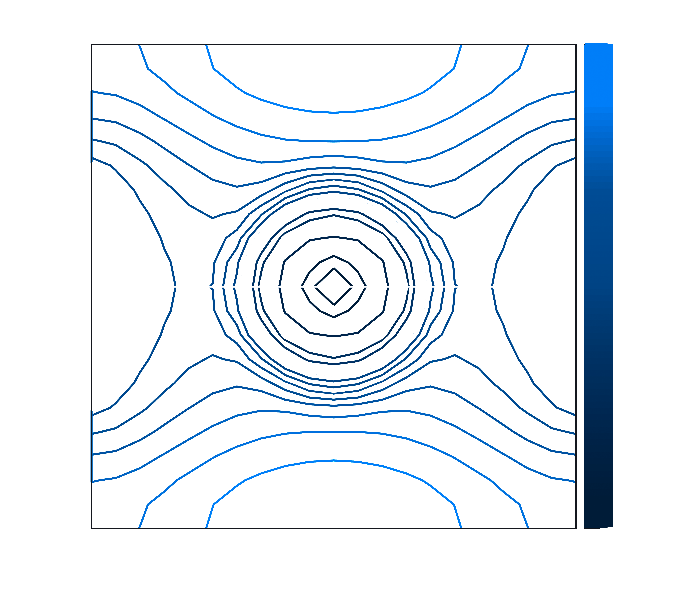


**Figure S2.** The dispersion curves. a) the dispersion curves of the Triangular lattice cell. b) the dispersion curves of the square lattice cell.

Section 4. Sound field distribution and FFT transformation

We simulated the acoustic pressure fields of heterogeneous bilayer metasurfaces at five twisting angles (0°, 26°, 45°, 60°, and 90°) under an excitation frequency of 1880 Hz using COMSOL Multiphysics. We also performed a two-dimensional FFT on the sound fields. Our findings indicate that as the twisting angle transitions from 0° to 90°, the propagation of acoustic pressure shifts from a clearly directional hyperbolic propagation to a divergent elliptical propagation. Notably, at 26°, the hybrid dispersion curve approaches flatness, resulting in highly directed sound propagation with minimal diffraction, thus achieving efficient transmission. It is worth noting that during the 360° rotation of one layer in the tHB system, there are four equivalent magic angles, namely $\theta$, $-\theta$，180$^{\circ}$-$\theta$ and 180$^{\circ}$+$\theta$. This arises from the inherent rotational symmetry of the metasurface, where specific angles produce identical effects.


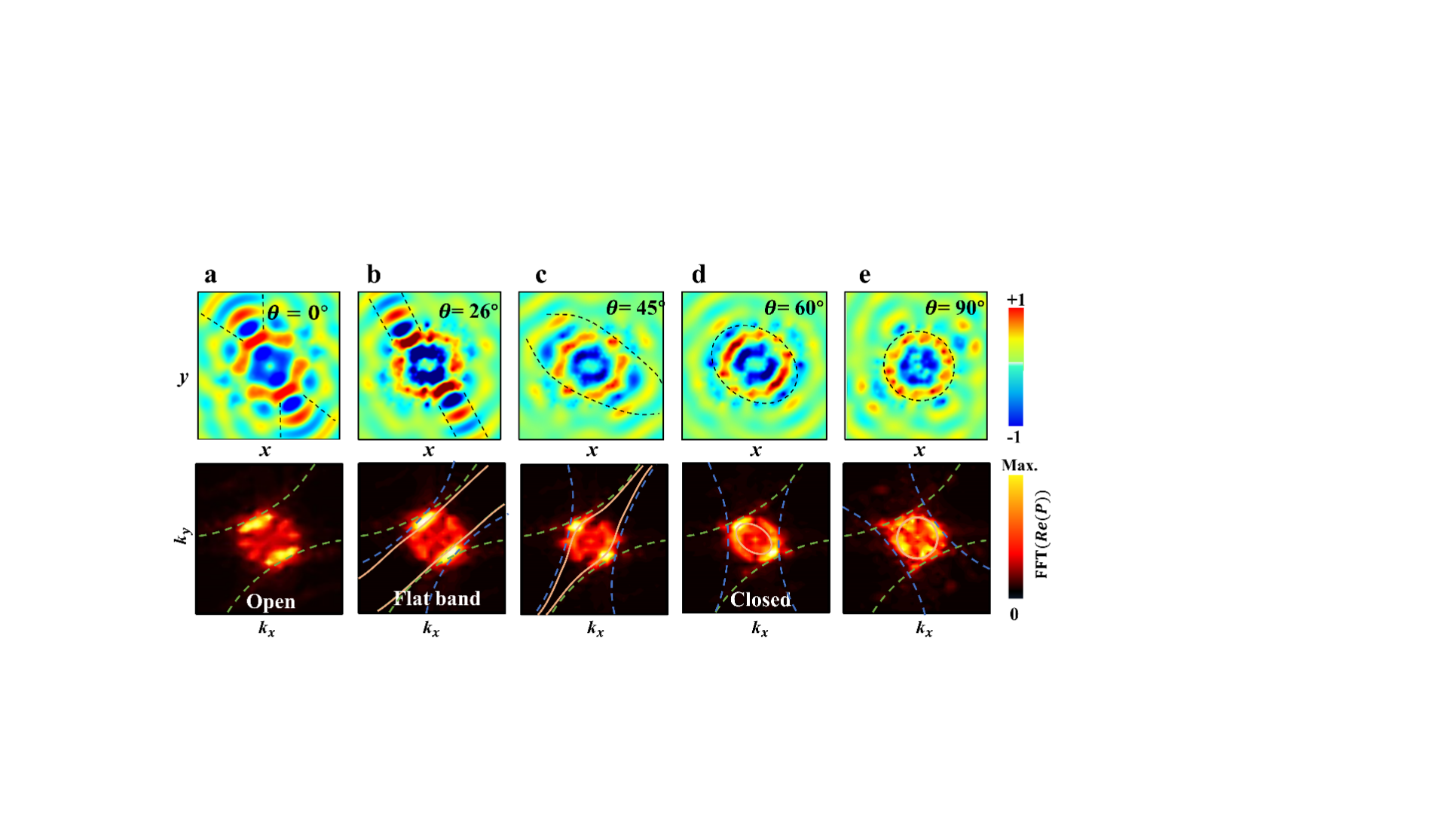


**Figure S3.** Sound field distribution and corresponding FFT transformation in the tHB system at *f* = 1880 Hz for different twisting angles. The blue and green dashed lines represent the dispersion lines of the tHB system, while the orange solid line indicates the dispersion curve calculated from theoretical model.

Section 5. Open angle of the dispersion curve.

When the frequency *f* = 1880 Hz, the dispersion curve is as shown in theFigure S5, the opening angles of the two layers are *θ*_1_=61° and *θ*=67°, respectively. When the twist angle between the two layers exceeds |180$^{\circ}$−*θ*_1_−*θ*_2_|, the moiré metasurface exhibits an elliptical mode; when the twist angle is smaller than |180$^{\circ}$−*θ*_1_−*θ*_2_|, it exhibits a hyperbolic mode. At this specific twist angle, the dispersion curve flattens, and the field becomes highly directional. Consequently, we term this angle the topological transition magic angle.


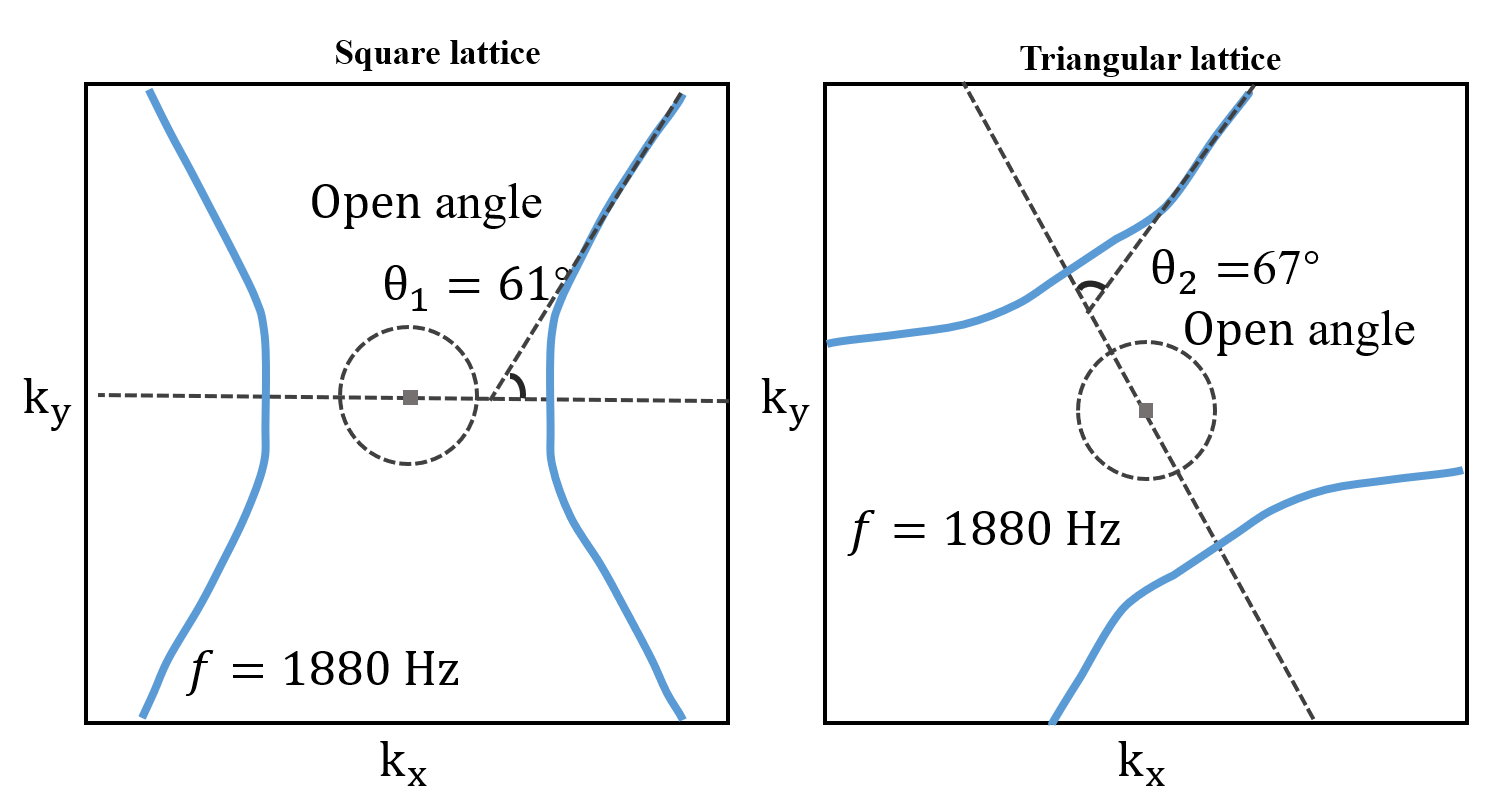


**Figure S4.** Dispersion curves of square and triangular lattices at *f*=1880 Hz.

Section 6. Magic angle and topological transitions.


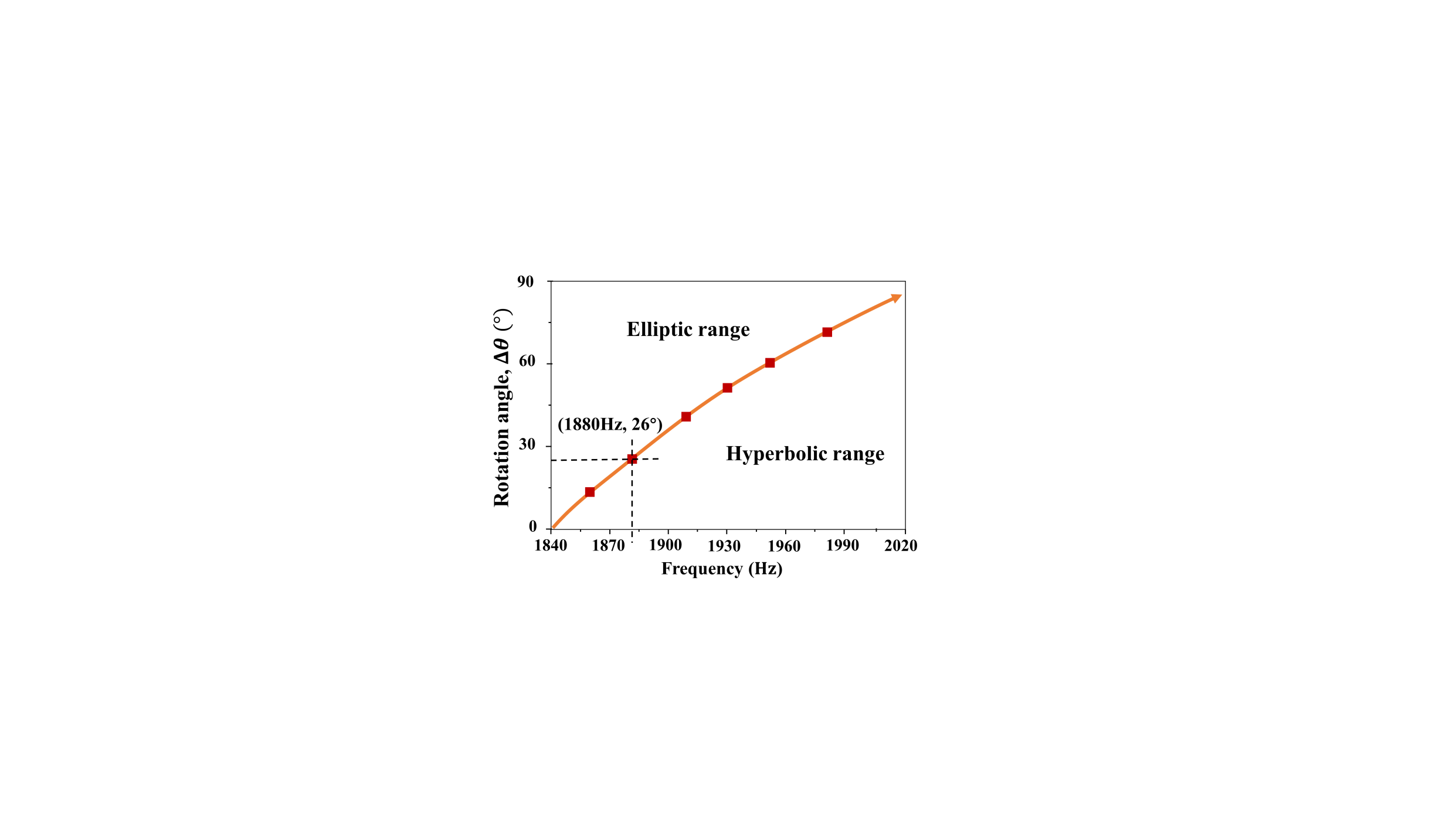


**Figure S5.** The relationship between frequency, twisting angle, and the orange arrow in the middle is used to distinguish between hyperbolic mode and elliptical mode.

Section 7. Energy distribution of sound propagation at different angles.

To better clarify this correlation of energy and the twist angle, we measured the energy distribution along the sound pressure propagation direction at various twisting angles. As shown in Figure S6, the orange line represents the simulation results, while the gray diamonds correspond to the experimental results, with both trends matching closely. We found that as the twisting angle increases from 0°, the energy transmission efficiency gradually increases, reaching its peak when the twisting angle reaches the magic angle of 26°. This is because at the magic angle, the dispersion curve hybridizes and flattens, enabling low-loss propagation. As the twisting angle continues to increase, energy transmission gradually decreases. In contrast, at a twisting angle of 90°, the hybridized dispersion curve closes, resulting in elliptical propagation. In this case, energy propagation lacks directionality, causing the energy transmission efficiency to drop to its lowest point.


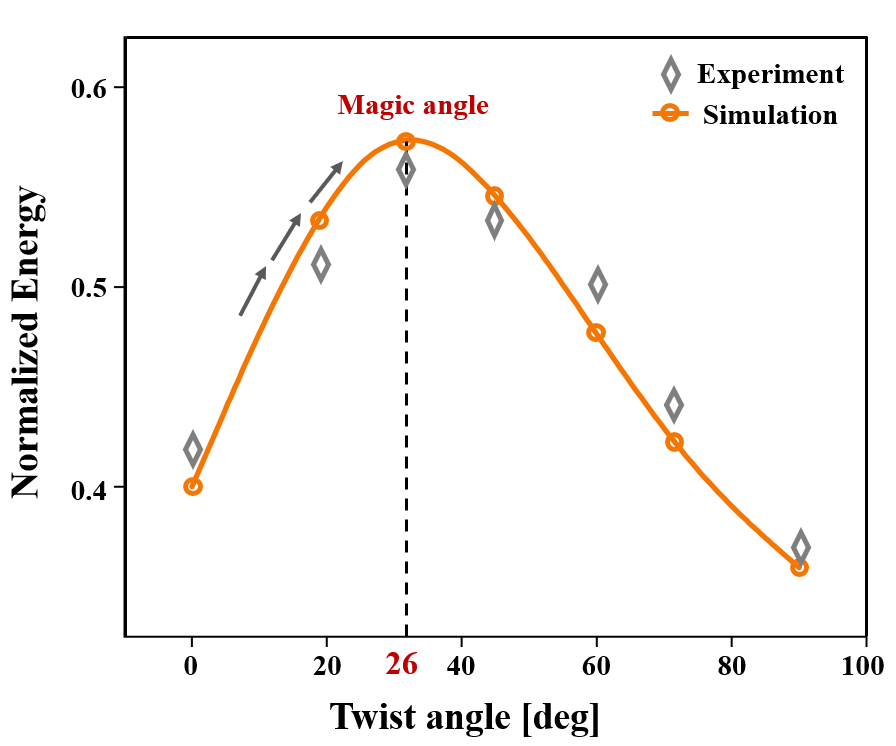


**Figure S6.** Energy distribution along the sound pressure propagation direction at different twist angles in the tHB system.

Section 8. The impact of interlayer distance on interlayer coupling.

We measured the sound pressure transmission rate of the tHB system at different interlayer coupling distances (0.3*a*_2_, 0.5*a*_2_, *a*_2_, 2*a*_2_, 3*a*_2_), as shown in the Figure S7. When the interlayer distance is greater than *a*_2_, the phenomenon becomes less pronounced, and the sound pressure transmission rate decreases more gradually.


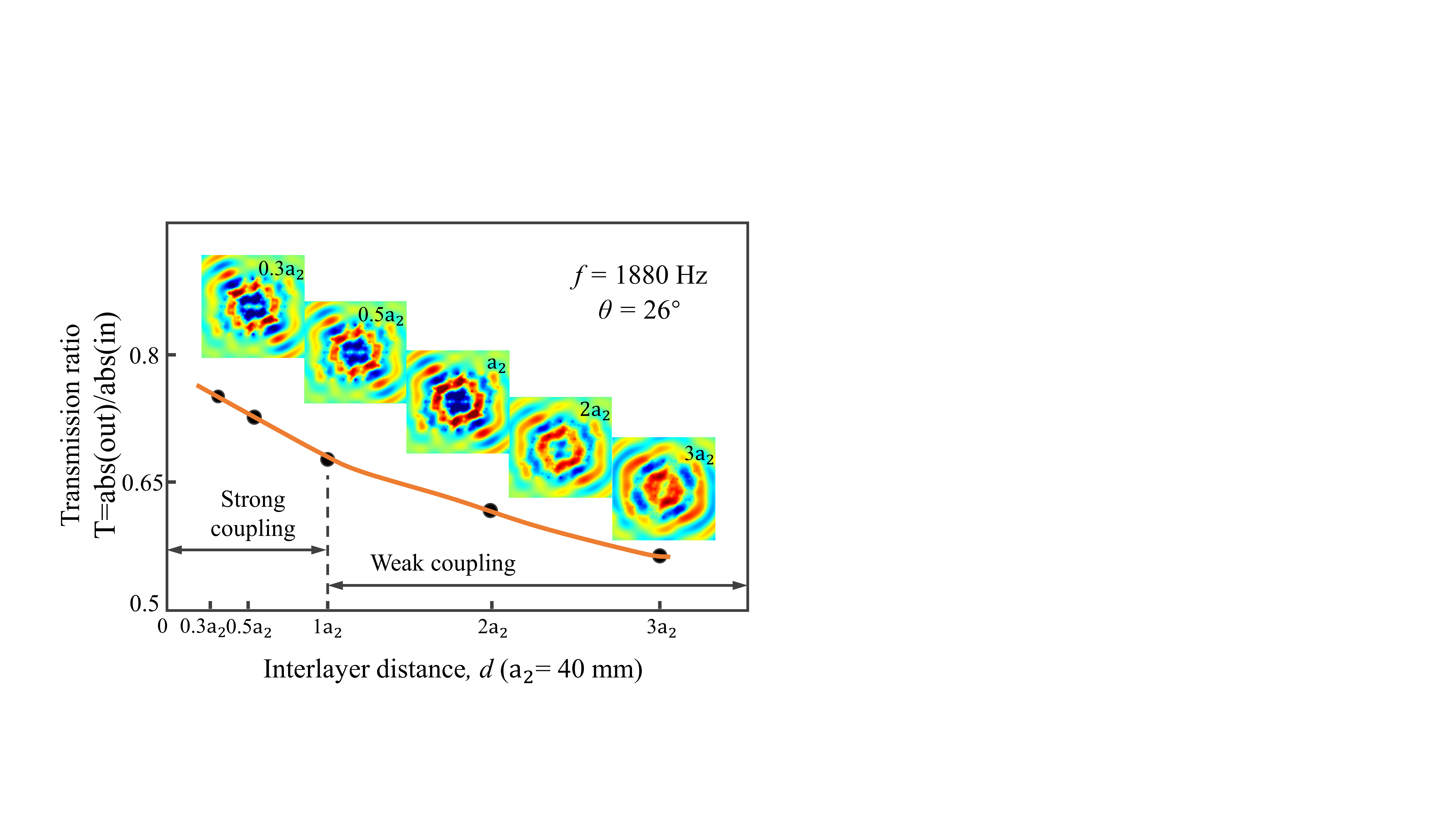


**Figure S7.** The impact of interlayer distance on the coupling effect of the tHB system. The sound pressure transmission rate were measured at different interlayer distances (0.3*a*_2_, 0.5*a*_2_, *a*_2_, 2*a*_2_, 3*a*_2_).
